# A review on the role of long non-coding RNA prostate androgen-regulated transcript 1 (PART1) in the etiology of different disorders

**DOI:** 10.3389/fcell.2023.1124615

**Published:** 2023-02-15

**Authors:** Soudeh Ghafouri-Fard, Atefeh Harsij, Bashdar Mahmud Hussen, Snur Rasool Abdullah, Aria Baniahmad, Mohammad Taheri, Guive Sharifi

**Affiliations:** ^1^ Department of Medical Genetics, School of Medicine, Shahid Beheshti University of Medical Sciences, Tehran, Iran; ^2^ Phytochemistry Research Center, Shahid Beheshti University of Medical Sciences, Tehran, Iran; ^3^ Department of Pharmacognosy, College of Pharmacy, Hawler Medical University, Erbil, Kurdistan Region, Iraq; ^4^ Medical Laboratory Science, Lebanese French University, Erbil, Kurdistan Region, Iraq; ^5^ Institute of Human Genetics, Jena University Hospital, Jena, Germany; ^6^ Urology and Nephrology Research Center, Shahid Beheshti University of Medical Sciences, Tehran, Iran; ^7^ Skull Base Research Center, Loghman Hakim Hospital, Shahid Beheshti University of Medical Sciences, Tehran, Iran

**Keywords:** lncRNA, PART1, cancer, biomarker, diagnsotic marker

## Abstract

LncRNA prostate androgen-regulated transcript 1 (PART1) is an important lncRNA in the carcinogenesis whose role has been firstly unraveled in prostate cancer. Expression of this lncRNA is activated by androgen in prostate cancer cells. In addition, this lncRNA has a role in the pathogenesis intervertebral disc degeneration, myocardial ischemia-reperfusion injury, osteoarthritis, osteoporosis and Parkinson’s disease. Diagnostic role of PART1 has been assessed in some types of cancers. Moreover, dysregulation of PART1 expression is regarded as a prognostic factor in a variety of cancers. The current review provides a concise but comprehensive summary of the role of PART1 in different cancers and non-malignant disorders.

## Introduction

Long non-coding RNAs (lncRNAs) have diverse roles in the carcinogenesis through modulation of gene expression. They can be localized in the nucleus or cytoplasm, thus regulating expression of genes through epigenetic, transcriptional and post-transcriptional mechanisms (Zhang et al., 2019a; Hussen et al., 2022). These effects are mediated through interactions with mRNAs, DNA molecules, proteins, and miRNAs (Zhang et al., 2019a; Ghafouri-Fard et al., 2022). The majority of identified lncRNAs are transcribed by RNA polymerase II; thus, they share several structural features with mRNAs, particularly in terms of having cap structure and poly A tail (Marchese et al., 2017). Yet, most lncRNAs lack coding capacity. The ENCODE project has annotated approximately 16,000 lncRNA genes in humans. These genes can produce more than 28,000 distinctive transcripts (Derrien et al., 2012).

LncRNAs have been shown to be involved in the carcinogenesis through modulation of expression of several tumor suppressor genes and oncogenes. Their altered expression in malignant cells have been associated with diverse abnormalities in the cell cycle regulation, cell proliferation, differentiation and apoptosis (Jiang et al., 2019). During the carcinogenesis process, lncRNAs regulate cell migration, invasion and stemness, thus they have prominent roles in the metastasis (Jiang et al., 2019).

**FIGURE 1:**
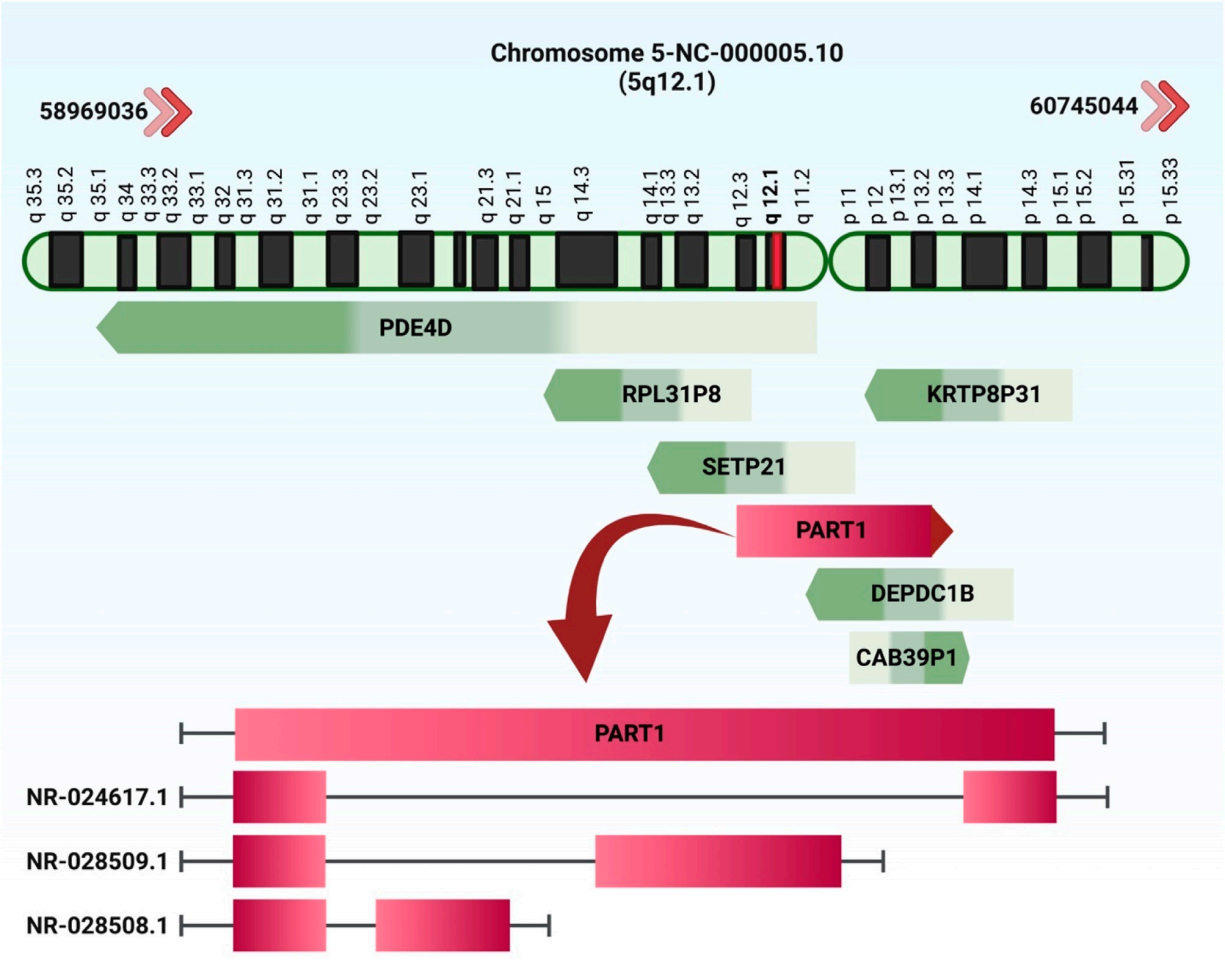
The chromosomal location of the prostate androgen-regulated transcript 1 (PART1) was initially identified in PCa. The NCBI database reveals the existence of three transcripts: NR 024617.1 (2.5 kb), NR 028509.1 (5.9 kb), and NR 028508.1 (2.1 kb).

LncRNA prostate androgen-regulated transcript 1 (PART1) is an important lncRNA in the carcinogenesis whose role has been firstly unraveled in prostate cancer. Expression of this lncRNA is activated by androgen in prostate cancer cells (Lin et al., 2000). Being encoded by a gene on gene to chromosome 5q12, PART1 has multiple alternatively transcripts none of them encoding a protein product (Figure 1). Expression assays have revealed biased expression of PART1 in brain, prostate, salivary gland, placenta and bladder (https://www.ncbi.nlm.nih.gov/gene/25859).

This lncRNA has dual roles in human tissues, being regarded as an oncogene in some tissues but tumor suppressor gene in others (Ran et al., 2022). The current review provides a concise but comprehensive summary of the role of PART1 in different cancers and non-malignant disorders.

## Role of PART1 in cancers

### Cell line studies

Functional studies in a variety of cancer-derived cell lines have assessed the consequences of up-regulation or silencing of PART1. Moreover, these studies have revealed a number of PART1 counterparts. In bladder cancer cells, enhanced expression of PART1 has promoted cell proliferation and invasiveness and suppressed cell apoptosis. On the other hand, PART1 silencing has suppressed cell proliferation and invasion and promoted apoptosis (Hu et al., 2019). In breast cancer cells, knockdown of PART1 has led to decreased proliferation, invasion and migration. Besides, miR-4516 has been found to be a direct counterpart of PART1. Suppression of miR-4516 has been found to rescue the effects of PART1 knockdown on breast cancer cells. Therefore, PART1 binding with miR-4516 promotes development of this type of cancer (Wang and Xu, 2020). Another study in breast cancer cells has shown that PART1 silencing improves the sensitivity of these cells to cisplatin, promotes cell apoptosis, and decreases expression proteins contributing in drug resistance (Lou et al., 2020). PART1 has also been found to be is enriched in triple negative breast cancer cells and in Aldefluor^high^ cancer stem cells. PART1 silencing in these cell lines has reduced cell proliferation, migration, and mammosphere forming ability. This lncRNA has been able to affect expression of several genes, including myosin-Va, MYO5A, zinc fingers and homeoboxes protein 2 and ZHX2. In addition, expression of miR-190a-3p, miR-937-5p, miR-22-5p, miR-30b-3p, and miR-6870-5p has been shown to be affected by PART1. PART1 has a direct interaction with miR-937-5p (Cruickshank et al., 2021).

PART1 has also been among lncRNAs being targeted by the tumor suppressor protein ΔNp63α in cervical cancer cells (Liu et al., 2020).

In colorectal cancer cells, three independent studies have shown possible mechanisms for contribution of PART1 in the carcinogenesis. First, PART1 has been shown to regulate this process through targeting miR-150-5p/miR-520h/CTNNB1 axis and inducing activity of Wnt/β-catenin pathway (Zhou et al., 2020a). Moreover, PART1 can function as a molecular sponge for miR-143 in these cells (Hu et al., 2017). Finally, through sponging miR-150-5p, PART1 can increase expression of LRG1 in colorectal cancer cells (Lou et al., 2020).

**FIGURE 2:**
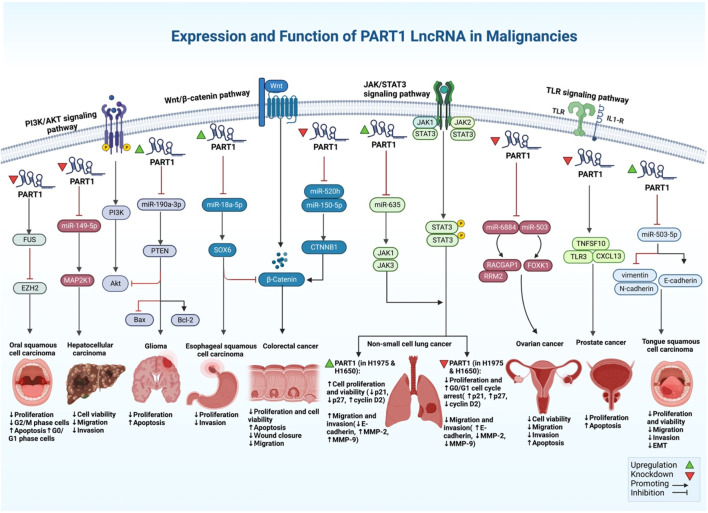
An illustration shows the different signaling pathways of PART1 lncRNA with its expression and function in different types of cancer.

In esophageal squamous cell carcinoma cells, PART1 has been shown to acts as a tumor suppressor lncRNA in a single study (Zhao et al., 2021). FOXP2 has been shown to bind to the promoter region of PART1 in these cells to regulate its expression. Up-regulation of PART1 could suppress cell proliferation and invasion, while its downregulation promotes cell proliferation and invasion in esophageal squamous cell carcinoma (Figure 2). From a mechanistical point of view, PART1 functions as a molecular sponge for miR-18a-5p, leading to over-expression of SOX6 and inactivation of the β-catenin/c-myc axis (Zhao et al., 2021). On the other hand, another study has shown that exosome-mediated transport of PART1 leads to induction of gefitinib resistance in esophageal squamous cell carcinoma cells through sponging miR-129 (Kang et al., 2018).

PART1 has been shown to restrain aggressive gastric cancer *via* decreasing expression of PDGFB through PLZF-mediated recruitment of EZH2 (Han et al., 2020). Similarly, PART1 has a tumor suppressor role in glioma through sponging miR-190a-3p and inactivating PTEN/AKT signals (Jin et al., 2020). Moreover, it can block carcinogenic process in glioma through modulation of miR-374b/SALL1 axis (Deng et al., 2022). Tables 1, 2 summarize the results of *in vitro* studies that reported up-regulation and down-regulation of PART1, respectively.

**TABLE 1:** *In vitro* experiments to examine expression and function of PART1 in malignancies in which PART1 has been up-regulated (TCLs: tumor cell lines, NCL: normal cell line, ∆: knockdown or deletion, EMT: epithelial-mesenchymal transition, Brdu: Bromodeoxyuridine, DDP: cisplatin, ↑: increase, ↓: decrease).

Tumor type	Cell line	Expression	Targets/Regulators and signaling pathways	Function	References
Bladder cancer	TCLs: 5637, T24	—	—	∆PART1: ↓proliferation, ↑apoptosis, ↓invasion	Hu et al. (2019)
Breast cancer	TCLs: MCF-7, SKBR3, BT-20, MDA-MB-231, ZR-75-1	Up (TCLs vs. NCLs)	miR-4516	∆PART1: ↓proliferation, ↓migration, ↓invasion	Wang and Xu (2020)
	NCL: MCF-10A				
	TCLs: MCF-7, T47D, MDA-MB-435, BT-549 NCL: MCF-10A	Up (TCLs vs. NCLs)	—	∆PART1: ↓proliferation (↓CDK2 and ↓cyclinE1, ↑P21), ↓migration and invasion (↓MMP3, ↓MMP10 and ↓MMP13), ↑cisplatin sensitivity	Lou et al. (2020)
				∆PART1 (in cisplatin-treated cells)	
				↑apoptosis (↑Bax and cleaved caspase-3, ↓Bcl-2)	
				∆PART1 (in cisplatin-resistant cells)	
				↓Chemo-resistance: ↓MDP1, ↓MRP1, ↓GST-π, ↓ABCB1 (chemoresistance proteins)	
Triple-negative breast cancer (TNBC)	TCLs: such as HCC1806, HCC1395	—	miRNAs-PART1 interactions→ gene expression alterations (genes like MYO5A, ZHX2, BICC1 and PPP2R3A)	∆PART1: ↓proliferation, ↓migration, ↓mammosphere formation ability, ↓MYO5A, ZHX2 and BICC1 expression (oncogenes), ↑PPP2R3A expression (tumor suppressor)	Cruickshank et al. (2021)
Colorectal cancer (CRC)	TCLs: HCT-116, SW116, SW480, HT29	Up (TCLs vs. NCLs)	miR-150-5p/miR-520h/CTNNB1, Wnt/β-catenin pathway	∆PART1: ↓proliferation and cell viability, ↑apoptosis, ↓wound closure, ↓migration	Zhou et al. (2020a)
	NCL: NCM460				
	TCLs: LoVo, HCT-116, SW620, SW480, HT29	Up (TCLs vs. NCLs)	miR-143/DNMT3A	∆PART1 (in SW620): ↓proliferation, ↓migration, ↓invasion	Hu et al. (2017)
	NCL: FHC			↑PART1 (in LoVo)	
				↑cell growth, ↑migration, ↑invasion	
	TCLs: HCT116, HT29, HEK-293T	—	miR-150-5p/LRG1	↑PART1 (in HCT116)	Lou et al. (2020)
				↑proliferation (↑Brdu + cells), ↑migration, ↓apoptosis, ↑EMT (↑vimentin, ↓E-cadherin)	
				∆PART1 (in HT29)	
				↓proliferation (↓Brdu + cells), ↓migration, ↑apoptosis, ↓EMT (↓vimentin, ↑E-cadherin)	
Hepatocellular Carcinoma (HCC)	TCLs: SK-HEP-1, Huh-7, Huh-1, Hep3B	Up (TCLs vs. NCLs)	miR-149-5p/MAP2K1	∆PART1: ↓cell viability, ↓migration, ↓invasion	Zhou et al. (2020b)
	NCL: THLE-2				
	TCLs: SMMC-7721, Huh-7	Up (TCLs vs. NCLs)	miR-590-3p/HMGB2	∆PART1: ↓proliferation, ↓colony formation, ↓invasion	Pu et al. (2020)
	NCL: LO2				
	TCLs: HB611, Huh7, HCCLM3, Bel-7405	Up (TCLs vs. NCLs)	miR-372-3p/TLR4	↑PART1	Zhou et al. (2022)
	NCL: THLE-2, THP-1			↑cell viability, ↑migration, ↑invasion, ↑EMT (↓E-cadherin, ↑N-cadherin, ↑vimentin, ↑Twist, ↑Snail)	
				↑M2 macrophage polarization (↑M2 macrophage markers (Arg-1 and IL-10), ↓M1 macrophage markers (iNOS and TNF-α))	
Liver cancer	TCLs: HepG2, HuH7, Hep3B	Up (TCLs vs. NCLs)	miR-3529-3p/FOXC2/AKT pathway (MMP-2 and MMP-9)	∆PART1: ↓cell viability, ↓migration, ↓invasion	Weng et al. (2021)
	NCL: LO2				
Lung Squamous Cell Carcinoma (LSCC)	TCLs: H2170, H226, H520, SK-MES-1	Up (TCLs vs. NCLs)	miR-185-5p/Six1	∆PART1 (in H2170)	Cao et al. (2021)
				↓colony formation ability, ↓cell viability, ↓migration, ↓invasion, ↓EMT (↑E-cadherin, ↓vimentin, ↓N-cadherin), ↑apoptosis	
	NCL: BEAS-2B			↑PART1 (in H520)	
				↑colony formation ability, ↑proliferation, ↑migration, ↑invasion, ↑EMT (↓E-cadherin, ↑vimentin, ↑N-cadherin), ↓apoptosis	
Non-small cell lung cancer (NSCLC)	TCLs: A549, H1650, H1975, SK-MES-1	Up (TCLs vs. NCLs)	miR-635/JAK1 and JAK3 (JAK/STAT3 signaling pathway)	↑PART1 (in H1975 & H1650)	Zhu et al. (2019)
				↑cell proliferation and viability (↓p21, ↓p27, ↑cyclin D2), ↑migration and invasion (↓E-cadherin, ↑MMP-2, ↑MMP-9)	
	NCL: BEAS-2B, HEK-293T			∆PART1 (in A549 & SK-MES-1)	
				↓proliferation and ↑G0/G1 cell cycle arrest (↑p21, ↑p27, ↓cyclin D2), ↓migration and invasion (↑E-cadherin, ↓MMP-2, ↓MMP-9)	
	TCLs: SPC-A1, H1299, A549, H1650, H1975, PC-9	Up (TCLs vs. NCLs)	miR-17-5p/TGFBETAR2	∆PART1: ↓proliferation, ↓migration, ↓invasion	Chen et al. (2021)
	NCL: 16HBE				
	TCLs: A549, NCI-H2444, NCI-H647, NCI-H23	Up (TCLs vs. NCLs)	—	∆PART1: ↑erlotinib sensitivity (in TCLs with wild-type *KRAS*)	Chen et al. (2020)
	NCL: BEAS-2B				
Oral Squamous Cell Carcinoma (OSCC)	TCLs: Tca-8113, CAL27	Up (TCLs vs. NCLs)	FUS/EZH2	∆PART1: ↓proliferation, ↓G2/M phase cells, ↑apoptosis, ↑G0/G1 phase cells	Yu et al. (2021)
	NCL: NHOK				
Ovarian Cancer (OC)	TCLs: CaoV-3, SK-OV-3, HO-8910	Up (TCLs vs. NCLs)	miR-503-5p/FOXK1	∆PART1: ↓viability, ↓migration, ↓invasion, ↑apoptosis	Li et al. (2022a)
	NCL: IOSE80				
	TCLs: Caov3, OVCAR3, A2780, SKOV3	Up (TCLs vs. NCLs)	miR-6884-5p/RACGAP1 and RRM2	∆PART1: ↓proliferation, ↓migration, ↓invasion	Li et al. (2022b)
	NCL: IOSE-386				
	TCLs: CAOV3, A2780 (DDP-resistant cell lines)	Up (DDP-resistant cell lines vs. control parental cell lines)	Transcriptional inducer of PART1: YY1	∆PART1 (in DDP-resistant cells)	Yang et al. (2021a)
			Targets of PART1: miR-512-3p/CHRAC1	↓proliferation, ↓migration, ↓invasion, ↑apoptosis, ↑chemosensitivity	
Pancreatic Cancer	TCLs: AsPC-1, Panc-1, SW 1990, BxPC-3	Up (TCLs vs. NCLs)	miR-122	∆PART1: ↓proliferation, ↓invasion, ↑apoptosis (↓Bcl-2, ↑Bax)	Ghafouri-Fard et al. (2021)
	NCL: HPDE6c7				
	TCL: PANC-1	—	hsa-mir-21/SCRN1	∆PART1: ↓proliferation, ↓migration	Lu et al. (2022)
Prostate cancer (PCa)	TCLs: LNCaP, PC3	—	Target: TLR signaling pathway (TLR3, TNFSF10, CXCL13)	∆PART1: ↓proliferation, ↑apoptosis	Sun et al. (2018)
			Transcriptional modulators of PART1: androgens		

**TABLE 2:** *In vitro* experiments to examine expression and function of PART1 in malignancies in which PART1 has been down-regulated (↑: increase, ↓: decrease).

Tumor type	Cell line	Expression	Targets/Regulators and signaling pathways	Function	References
Cervical Squamous Cell Carcinoma (CSCC)	TCLs: SiHa, ME-180, C-33A, HeLa, HaCat, 293T	_	Transcriptional regulator of PART1: ∆Np63α	↑PART1 (in SiHa)	Liu et al. (2020)
				↓proliferation and colony formation, ↓S phase cells, ↑G1 phase cells, ↓migration, ↓invasion	
				∆PART1 (in ME-180)	
				↑proliferation and colony formation, ↑S phase cells, ↓G1 phase cells, ↑migration, ↑invasion	
Esophageal Squamous Cell Carcinoma (ESCC)	TCLs: Eca109, EC9706, TE1, KYSE70, KYSE450	Down (TCLs vs. NCLs)	Regulator: FOXP2	↑PART1	Zhao et al. (2021)
	NCL: Het-1A		Target: miR-18a-5p/SOX6/β-catenin signaling pathway	↓proliferation, ↓invasion	
	TCLs: TE1, TE6, TE8, TTn, KYSE-450 (gefitinib resistant cell lines)	Up (gefitinib resistant cell lines vs. parental cell lines)	Transcriptional inducer of PART1 in gefitinib resistant cells: STAT1	∆PART1	Kang et al. (2018)
			Targets of PART1: miR-129, Bcl-2/Bax signaling pathway	↑gefitinib chemotoxicity, ↑cell apoptosis	
Gastric cancer (GC)	TCLs: MGC-803, BGC-823, SGC-7901, NCI-N87, AGS, NUGC-3	Down (TCLs vs. NCLs)	AR/PLZF/EZH2/PDGFB → PDGFRβ/PI3K/Akt signaling pathway	↑PART1 (in MGC-803, BGC-823, SGC-7901)	Han et al. (2020)
				↓cell viability and colony formation, ↓migration, ↓invasion	
	NCL: GES-1			∆PART1 (in AGS)	
				↑cell viability and colony formation, ↑migration, ↑invasion	
Glioma	TCLs: U87MG, LN-18, LN-428	Down (TCLs vs. astrocytes)	miR-190a-3p, PTEN, PI3K/AKT signaling pathway	↑PART1	Jin et al. (2020)
				↓proliferation, ↑apoptosis (↓Bcl-2, ↑Bax)	
	TCLs: A172, U373, LN229, U251	Down (TCLs vs. NCLs)	miR-374b/SALL1	↑PART1	Deng et al. (2022)
	NCL: NHA			↓cell proliferation and viability, ↓migration, ↓EMT (↑E-cadherin, ↓N-cadherin, vimentin and Snail)	
Head and Neck Squamous Cell Carcinoma (HNSCC)	TCLs: CNE-2, C666-1, SCC-4	Down (TCLs vs. NCLs)	—	—	Yang et al. (2021b)
	NCL: HOK, NP69				
Tongue Squamous Cell Carcinoma (TSCC)	TCLs: CAL-27, SCC9, SCC25	Down (TCLs vs. NCLs)	miR-503-5p	↑PART1	Liu et al. (2020)
	NCL: NHOK			↓proliferation and viability, ↓migration, ↓invasion, ↓EMT (↓N-cadherin, ↓vimentin, ↑E-cadherin)	

### Animal studies

**FIGURE 3:**
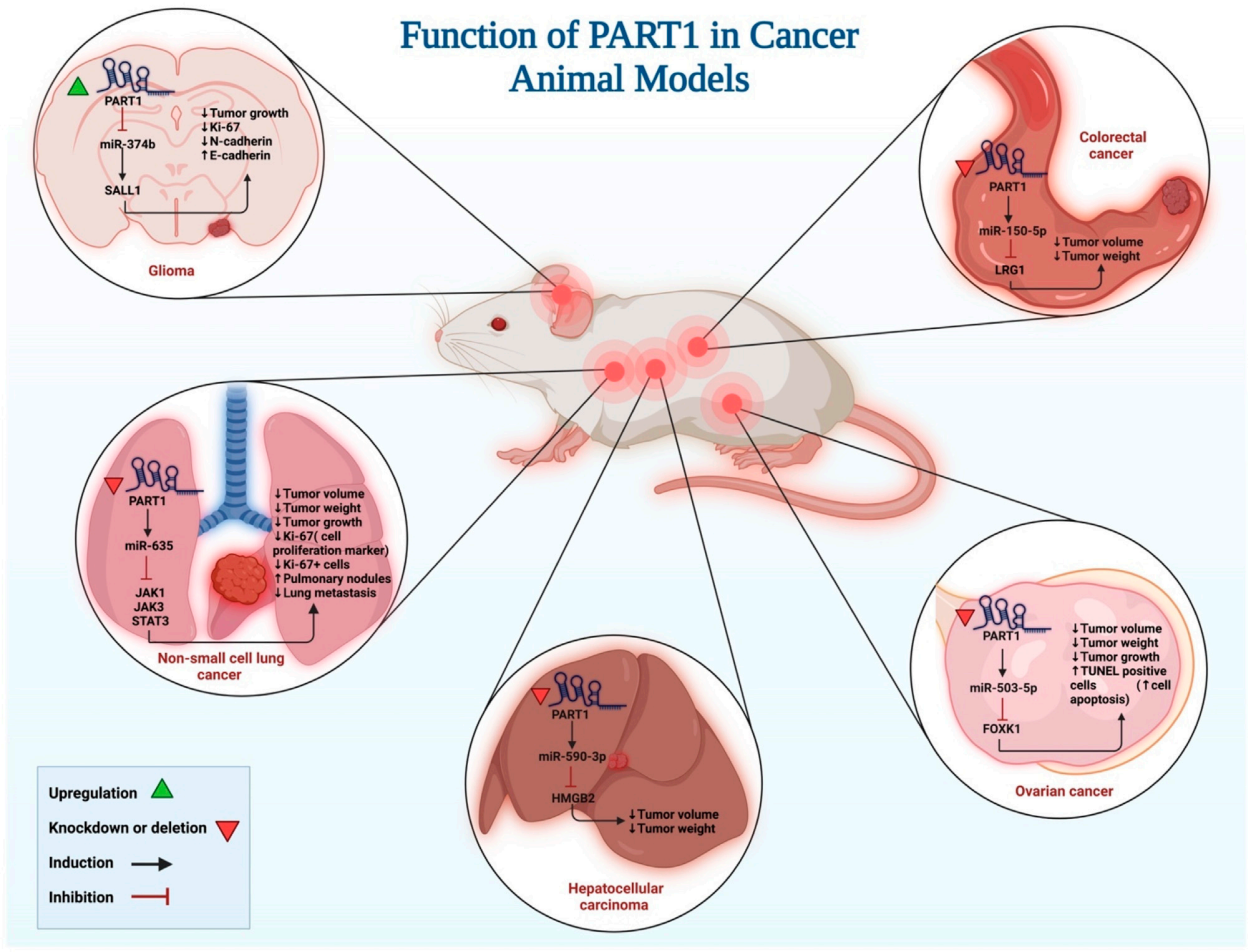
An illustration depicts the roles of PART1 activation and silencing in tumor formation in xenograft models, as well as the signaling pathways involved.

Different study groups have evaluated functional consequences of PART1 up-regulation or silencing on tumor formation in xenoraft models (Figure 3) (Table 3). Similar to *in vitro* studies, both tumor suppressor role and oncogenic role have been reported for PART1. Examples of the former type of function have been seen in animal models of cervical squamous cell carcinoma (Liu et al., 2020), gastric cancer (Han et al., 2020) and glioma (Deng et al., 2022) where up-regulation of PART1 has resulted in reduction of tumor growth. Animal models of colorectal carcinoma (Zhou et al., 2020a), hepatocellular carcinoma (Pu et al., 2020), lung cancer (Zhu et al., 2019), oral squamous cell carcinoma (Yu et al., 2021), ovarian cancer (Li et al., 2022b) and triple negative breast cancer (Cruickshank et al., 2021).

**TABLE 3:** Effects of PART1 in animal models for cancer (∆: knockdown or deletion, NR: not reported, CAM: chorioallantoic membrane, NOD-SCID mice: non-obese diabetic-severe combined immunodeficiency mice, TUNEL: terminal deoxynucleotidyl transferase dUTP nick end labeling, SPF: specific-pathogen-free, ↑: increase, ↓: decrease).

Tumor type	Animal models (experimental and control group)/Number of studied animals	Target	Function	References
Cervical Squamous Cell Carcinoma (CSCC)	Female athymic nude mice/10 (5 for each group)	NR	↑PART1	Liu et al. (2020)
			↓tumor growth	
Colorectal cancer (CRC)	BALB/c nude mice/NR	∆PART1	∆PART1	Zhou et al. (2020a)
		↑miR-150-5p and miR-520 h	↓tumor volume, ↓tumor weight, ↓tumor growth, ↓Ki-67, ↓β-catenin, ↓PCNA, ↓vimentin	
	BALB/c nude mice/NR	NR	∆PART1	Hu et al. (2017)
			↓tumor growth, ↓tumor size, ↓tumor volume	
	Male BALB/c nude mice/10 (5 for each group)	∆PART1	∆PART1	Lou et al. (2020)
		↑miR-150-5p, ↓LRG1	↓tumor volume, ↓tumor weight	
Esophageal Squamous Cell Carcinoma (ESCC)	Male BALB/c nude mice/NR	NR	↑PART1	Kang et al. (2018)
			↑gefitinib resistance, ↑Bcl-2, ↓Bax, ↓cleaved caspase-3, ↓cleaved PARP	
Gastric cancer	Chick embryo CAM/NR	NR	↑PART1	Han et al. (2020)
			↓tumor weight, ↓metastatic tumor colonies, ↓human Alu expression	
			∆PART1	
			↑tumor weight, ↑lung metastasis	
	NOD-SCID mice/6 (3 for each group)	NR	↑PART1	Han et al. (2020)
			↓tumor growth, ↓lung metastasis, ↓human Alu expression	
			∆PART1	
			↑tumor weight, ↑lung metastasis	
Glioma	SPF-grade nude mice/8 (4 for each group)	↑PART1	↑PART1	Deng et al. (2022)
		↑SALL1, ↓miR-374b	↓tumor growth, ↓Ki-67, ↓N-cadherin, ↑E-cadherin	
Hepatocellular Carcinoma (HCC)	BALB/c nude mice/NR	∆PART1	∆PART1	Pu et al. (2020)
		↓HMGB2, ↑miR-590-3p	↓tumor volume, ↓tumor weight	
	Male nude mice/12 (6 for each group)	NR	∆PART1: ↓tumorigenicity	Zhou et al. (2022)
			↓tumor size, ↓tumor volume, ↓tumor mass, ↓Ki-67 positive cells, ↑apoptosis, ↑E-cadherin, ↓N-cadherin, ↓Twist, ↓Snail	
Non-small cell lung cancer (NSCLC)	BALB/c nude mice/NR	∆PART1	∆PART1	Zhu et al. (2019)
		↑miR-635, ↓JAK1, JAK3 and STAT3	↓tumor volume, ↓tumor weight, ↓tumor growth, ↓Ki-67 (cell proliferation marker), ↓Ki-67 + cells, ↑pulmonary nodules, ↓lung metastasis	
	Female athymic BALB/c nude mice/15	NR	∆PART1	Chen et al. (2021)
			↓tumor volume, ↓tumor weight, Ki-67 positive cells	
Oral Squamous Cell Carcinoma (OSCC)	BALB/c nude mice/16 (8 for each group)	∆PART1	∆PART1	Yu et al. (2021)
		↓EZH2	↓tumor volume, ↓PCNA, ↓cyclinD1, ↓Bcl-2, ↑Bax, ↑cleaved caspase-3	
Ovarian Cancer (OC)	BALB/c nude mice/10 (5 for each group)	∆PART1	∆PART1	Li et al. (2022a)
		↓FOXK1, ↑miR-503-5p	↓tumor volume, ↓tumor weight, ↓tumor growth, ↑TUNEL positive cells (↑cell apoptosis)	
	BALB/c nude mice/NR	NR	∆PART1	Li et al. (2022b)
			↓tumor volume, ↓tumor weight, ↓tumor growth	
Triple-negative breast cancer (TNBC)	NOD-SCID female mice/14 (7 for each group)	NR	∆PART1	Cruickshank et al. (2021)
			↓tumor volume, ↓tumor weight, ↓mammosphere formation ability	

### Studies in clinical samples

Studies in clinical samples have shown up-regulation of PART1 in a variety of cancer tissues including bladder, breast and colorectal cancers (Tables 4, 5). However, there are a number of other cancerous tissues in which PART1 has been found to be down-regulated. For instance, expression of PART1 has been shown to be decreased in esophageal squamous cell carcinoma tissues parallel with down-regulation of SOX6. Notably, low expression of these two genes has been associated with TNM stage, lymph node metastasis and poor prognosis in these patients. Moreover, expression of FOXP2 has been reduced in these tissues in correlation with PART1 expression levels (Zhao et al., 2021). However, another study in this type of cancer has revealed up-regulation of PART1 in the sera samples of gefitinib non-responders *versus* responders (Kang et al., 2018). Moreover, PART1 is down-regulated in cervical squamous cell carcinoma tissues (Liu et al., 2020). In addition, dysregulation of PART1 has been associated with TNM stage, metastasis, tumor grade and diameter as well as histological type in a variety of cancers (Tables 4, 5).

**TABLE 4:** Function of PART1 up-regulation in the development of malignancy on the basis of studies in clinical samples (ANTs: adjacent normal tissues, TCGA: the cancer genome atlas, METABRIC: molecular taxonomy of breast cancer international consortium, GEPIA: gene expression profiling interactive analysis, GEO: gene expression omnibus, GTEx: genotype–tissue expression, ENCORI: encyclopedia of RNA interaction, GBM: high-grade glioma, LGG: low-grade glioma, ER: early recurrence, BCLC: Barcelona clinic liver cancer, OS: overall survival, DFS: disease-free survival, FIGO: international federation of gynecology and obstetrics, TNM: tumor-node-metastasis, T stage: tumor stage, T classification: tumor classification).

Tumor type	Samples	Expression (tumor vs. normal control)	Kaplan-meier analysis	Univariate cox regression analysis	Multivariate cox regression analysis	Association of dysregulation of PART1 with clinicopathologic characteristics	References
Bladder Cancer	30 pairs of tumor tissues and ANTs + GEO database	Up-regulated	—	—	—	—	Hu et al. (2019)
Breast Cancer	31 pairs of tumor tissues and ANTs	Up-regulated	High PART1 expression correlated with poorer OS	—	—	Metastasis, tumor stage	Wang and Xu (2020)
	30 pairs of tumor tissues and ANTs	Up-regulated	—	—	—	—	Lou et al. (2020)
Triple-negative breast cancer (TNBC)	Datasets from METABRIC, Cell 2015 and TCGA PanCancer	Up-regulated (basal-like and TNBC vs. other subtype tumors)	High PART1 expression correlated with poorer survival (in basal-like BC)	—	—	—	Cruickshank et al. (2021)
Luminal Breast Cancer	10 pairs of tumor tissues and ANTs + TCGA data	Up-regulated	High PART1 expression correlated with poorer OS	—	—	Ki-67, tumor grade, tumor diameter	Jiang et al. (2020)
Clear cell Renal Cell Carcinoma (ccRCC)	254 tumor samples and 71 normal controls (from TCGA database)	_	Low PART1 expression correlated with longer OS	—	—	Tumor metastasis	Liu et al. (2019)
Colorectal Cancer (CRC)	38 pairs of tumor tissues and ANTs	Up-regulated	—	—	—	—	Zhou et al. (2020a)
	50 pairs of tumor tissues and ANTs	Up-regulated	High PART1 expression correlated with poorer OS	—	—	Tumor invasion, TNM stage	Hu et al. (2017)
	56 pairs of tumor tissues and ANTs	Up-regulated	—	—	—	Lymph node metastasis, invasion depth, TNM stage	Lou et al. (2020)
	10 patient blood samples and 10 normal blood samples	Up-regulated	—	—	—	—	Lou et al. (2020)
Esophageal Squamous Cell Carcinoma (ESCC)	79 serum samples from patients receiving gefitinib therapy (42 responding patients and 37 non-responding patients)	Up-regulated (non-responding vs. responding samples)	—	—	—	—	Kang et al. (2018)
Hepatocellular Carcinoma (HCC)	48 pairs of tumor tissues and ANTs	Up-regulated	—	—	—	—	Zhou et al. (2020b)
	374 tumor and 50 normal samples (from ENCORI website)	Up-regulated	—	—	—	—	Pu et al. (2020)
	255 HCC patients: 133 ER and 92 non-ER patients (from TCGA)	Up-regulated (ER vs. non-ER)	—	—	—	—	Lv et al. (2018)
	51pairs of tumor tissues and ANTs + TCGA data	Up-regulated	High PART1 expression correlated with poorer OS and DFS	—	—	—	Zhou et al. (2022)
Liver Cancer	30 patient blood samples and 30 normal blood samples	Up-regulated	—	—	—	Tumor size, TNM stage, BCLC stage	Weng et al. (2021)
Lung Squamous Cell Carcinoma (LSCC)	51 pairs of tumor tissues and ANTs	Up-regulated	High PART1 expression correlated with poorer OS	—	↑PART1, ↓miR-185-5P, ↑Six1, differentiation, lymph node metastasis (independent risk factors for OS)	Tumor size, histological stage, lymph node metastasis, differentiation	Cao et al. (2021)
Non-small cell lung cancer (NSCLC)	60 pairs of tumor tissues and ANTs	Up-regulated	High PART1 expression correlated with poorer OS	Histology and EGFR mutation (shorter OS)	PART1 expression, histology (independent prognostic factors for OS)	Histologic type (↑PART1 in squamous NSCLC tumors)	Zhu et al. (2019)
	208 pairs of tumor tissues and ANTs	Up-regulated	High PART1 expression correlated with poorer OS and DFS	High PART1 expression, high T stage, lymph node metastasis, poor differentiation (poor OS and DFS)	PART1 expression (independent prognostic factor for OS and DFS)	Histologic type (↑PART1 in squamous tumors)	Li et al. (2017)
	30 pairs of tumor tissues and ANTs	Up-regulated	—	—	—	—	Chen et al. (2021)
Oral Squamous Cell Carcinoma (OSCC)	36 pairs of tumor tissues and ANTs	Up-regulated	—	—	—	Tumor size, node metastasis, clinical stage	Yu et al. (2021)
Ovarian Cancer (OC)	50 pairs of tumor tissues and ANTs	Up-regulated	—	—	—	Lymph node metastasis, FIGO stage	Li et al. (2022a)
	426 tumor samples and 88 normal samples (from GEPIA)	Up-regulated	—	—	—	—	Li et al. (2022b)
	TCGA datasets	Up-regulated	—	—	—	—	Yang et al. (2021a)
Pancreatic Cancer	45pairs of tumor tissues and ANTs	Up-regulated	High PART1 expression correlated with poorer 5-year OS	—	—	Tumor size, T classification, clinical stage, vascular invasion	Ghafouri-Fard et al. (2021)
Pancreatic Neuroendocrine Tumors (PanNETs)	17 tumor tissues and 8 ANTs	Up-regulated	—	—	—	—	Xiao et al. (2019)
Prostate Cancer (PCa)	30 pairs of tumor tissues and ANTs	Up-regulated	—	—	—	Tumor stage, Gleason score	Sun et al. (2018)
	27 pairs of tumor tissues and ANTs	Up-regulated (in 18 patients), Down-regulated (in 7 patients), Similar expression (in 2 patients)	—	—	—	—	Sidiropoulos et al. (2001)

**TABLE 5:** Function of PART1 down-regulation in the development of malignancy on the basis of studies in clinical samples.

Tumor type	Samples	Expression (tumor vs. normal control)	Kaplan-meier analysis	Univariate cox regression analysis	Multivariate cox regression analysis	Association of dysregulation of PART1 with clinicopathologic characteristics	References
Cervical Squamous Cell Carcinoma (CSCC)	15 samples: 5 cervical cancer and 10 uterine myoma	Down-regulated (tumor vs. normal tissues)	—	—	—	—	Liu et al. (2020)
Esophageal Squamous Cell Carcinoma (ESCC)	75 pairs of tumor tissues and ANTs + TCGA database and GEO dataset	Down-regulated	Low PART1 expression correlated with shorter survival	—	—	TNM stage, lymph node metastasis	Zhao et al. (2021)
Gastric Cancer	15 pairs of tumor tissues and ANTs	Down-regulated	—	—	—	—	Gu et al. (2019)
	136 tumor tissues and 94 ANTs	Down-regulated	Low PART1 expression correlated with shorter OS	—	—	Distant tumor metastasis, liver metastasis	Han et al. (2020)
Glioma	50 tumor tissues and 6 normal brain tissues	Down-regulated	—	—	—	—	Jin et al. (2020)
	GEPIA and TCGA dataset	Down-regulated (GBM vs. normal, LGG vs. normal, and GBM vs. LGG)	—	—	—	—	Jin et al. (2020)
	665 tumor samples (from TCGA) and 188 normal control samples (from GTEx)	Down-regulated	—	—	—	—	Yang et al. (2021c)
Head and Neck Squamous Cell Carcinoma (HNSCC)	10 patient blood samples and 10 normal blood samples + GEPIA database	Down-regulated	—	—	—	—	Yang et al. (2021b)
Tongue Squamous Cell Carcinoma (TSCC)	40 pairs of tumor tissues and ANTs	Down-regulated	Low PART1 expression correlated with poorer OS	—	—	Tumor classification, clinical stage, lymph node metastasis	Liu et al. (2020)
	147 tumor samples and 15 normal samples (from TCGA database)	_	High PART1 expression correlated with longer OS	—	—	—	Song et al. (2019)
	122 tumor samples and 15 normal samples (from TCGA database)	Down-regulated	—	—	—	—	Zhang et al. (2019b)

### Diagnostic value of PART1

Diagnostic value of PART1 has been evaluated in the context of esophageal squamous cell carcinoma (Kang et al., 2018) and lung squamous cell carcinoma (Cao et al., 2021) (Table 6). In the former type of cancer, PART1 levels could differentiate between gefitinib responders and non-responders with AUC value of 0.839 (Kang et al., 2018). In the latter type of cancer, this lncRNA could separate cancerous and non-cancerous tissues with AUC value of 0.7857 (Cao et al., 2021).

**TABLE 6:** Value of PART1 in cancer diagnosis (ANTs: adjacent normal tissues).

Tumor type	Samples	Distinguish between	Area under the curve (AUC)	Sensitivity (%)	Specificity (%)	References
Esophageal Squamous Cell Carcinoma (ESCC)	79 serum samples from patients receiving gefitinib therapy	37 non-responding patients vs. 42 responding patients	0.839	78.6	86.5	Kang et al. (2018)
Lung Squamous Cell Carcinoma (LSCC)	51 pairs of tumor tissues and ANTs	LSCC tissues vs. ANTs	0.7857	66.67	86.27	Cao et al. (2021)

## Role of PART1 in non-malignant disorders

### Cell line studies

PART1 is among lncRNAs that are dysregulated in SARS-CoV-2 infected cells as revealed by an *in silico* analysis of GSE147507 dataset. Expression of PART1 has been found to reduced in at least two independent SARS-CoV-2-infected cell lines. Dysregulated lncRNAs have been shown to interact with a variety of genes/proteins and miRNAs which have been linked with signaling pathways regulating viral infection, inflammatory responses and immune function (Laha et al., 2021). PART1 is alos involved in the pathogenesis of intervertebral disc degeneration *via* regulation of the miR-93/MMP2 axis (Gao et al., 2020) as well as miR-190a-3p expression (Zhang et al., 2021a). Tables 7, 8 summarize the role of PART1 in other non-malignant disorders based on cell line studies that reported up-regulation and down-regulation of PART1, respectively.

**TABLE 7:** Cell line studies on PART1 function in non-malignant illnesses in which PART1 has been up-regulated (∆: knockdown or deletion, NP cells: nucleus pulposus cells, ECM: extracellular matrix, LPS: lipopolysaccharide, MPP+: methyl-4-phenyl-1, 2, 3, 6-tetrahydropyridine, H/R: hypoxia/reoxygenation, ROS: reactive oxygen species, MMP13: matrix metallopeptidase13, MMP: mitochondrial membrane potential, ↑: increase, ↓: decrease).

Disorder	Cell line	Expression	Targets/Regulators and signaling pathways	Function	References
Intervertebral Disc Degeneration (IDD)	NP cells (derived from IDD patients)	—	miR-93-5p/MMP2	∆PART1	Gao et al. (2020)
				↑proliferation (↑Ki-67), ↑colony formation ability, ↓apoptosis (↓cleaved caspase-3), ↑ECM synthesis (↑aggrecan and collagen II), ↓ECM degradation (↓ADAMTS4 and MMP13)	
	*In vitro* IDD models: LPS-stimulated NP cells	High (LPS-induced NP cells vs. normal NP cells)	miR-190a-3p	∆PART1	Zhang et al. (2021a)
	Controls: NP cells			↑cell viability, ↓apoptosis, ↓inflammatory response (↓TNF-α, ↓IL-1β, ↓IL-6), ↓ECM degradation (↑aggrecan, ↑collagen II)	
Osteoarthritis (OA)	C20/A4 (the immortalized human chondrocytes cell lines)	—	miR-590-3p, TGFBR2/Smad3 signaling pathway	∆PART1	Lu et al. (2019)
				↓cell viability, ↑apoptosis (↑cleaved caspase-3 and caspase-9, ↑Bax)	
				↑PART1	
				↓effects of IL-1β	
				↑cell viability and ↓apoptosis rate	
	OA chondrocytes and normal chondrocytes	High (OA cells vs. normal cells)	miR-373-3p/SOX4	∆PART1	Zhu and Jiang (2019)
				↓Cell proliferation and viability, ↓ECM degradation (↓MMP13, ↑collagen II, ↑aggrecan), ↑apoptosis (↓Bcl-2, ↑Bax, ↑cleaved caspase-3)	
Osteoporosis (OP)	hBMSCs (human bone marrow-derived mesenchymal stem cells)	High (osteogenesis-induced BMSCs vs. controls)	Targets: miR-185-5p/RUNX3	∆PART1 (in hBMSCs)	Zhang et al. (2021b)
			Transcriptional activator of PART1: RUNX3	↓osteogenic differentiation (↓osteogenesis markers such as OCN, OSX and COL1A, ↓ALP activity, ↓matrix mineralization), ↑apoptosis	

**TABLE 8:** Non-malignant illnesses in which PART1 has been down-regulated (↑: increase, ↓: decrease).

Disorder	Cell line	Expression	Targets/Regulators and signaling pathways	Function	References
COVID-19 (coronavirus disease 19)	A549, Calu3	Down-regulated (SARS-CoV-2 infected cells vs. control cells)	—	—	Laha et al. (2021)
Myocardial Ischemia-Reperfusion Injury (MI/RI)	*In vitro* H/R model: H/R NMVCs (neonatal mice ventricle cells)	Down-regulated (H/R cells vs. controls)	miR-503-5p/BIRC5	↑PART1	Guo et al. (2021)
	Controls: NMVCs			↑cell viability, ↓apoptosis (↓ H/R injury)	
				↑mitochondrial function (↓ROS, ↑ATP level, ↑GSH level, ↑MMP level)	
Parkinson’s disease (PD)	*In vitro* PD models: MPP(+)-treated SH-SY5Y cells	Down-regulated (PD model cells vs. controls)	microRNA-106b-5p/MCL1	↑PART1: ↓effects of MPP + treatment	Shen et al. (2021)
	Control group: SH-SY5Y cells			↑cell viability, ↓apoptosis (↓cleaved caspase-3), ↓inflammatory response (↓TNF-α, IL-1β and IL-6), ↓oxidative stress (↓LDH and ROS, ↑SOD)	

### Animal studies

Two different studies in animal models have shown the importance of PART1 in myocardial ischemia-reperfusion injury (Guo et al., 2021) and Parkinson’s disease (Shen et al., 2021) (Table 9). In animal models of myocardial ischemia-reperfusion injury, up-regulation of PART1 has resulted in the alleviation of tissue injury, enhancement of cardiac function and reduction of infarction size (Guo et al., 2021).

**TABLE 9:** Animal studies on the involvement of PART1 in non-malignant disorders (MPTP: methyl-4-phenyl-1, 2, 3, 6-tetrahydropyridine hydrochloride, I/R: Ischemia-Reperfusion, EF: ejection fraction, FS: fraction shortening, ↑: increase, ↓: decrease).

Disorder	Animal model (experimental and control group)/Number of studied animals	Expression	Result	References
Myocardial Ischemia-Reperfusion Injury (MI/RI)	Male C57BL/6 mice (*in vivo* I/R model)/40	Down-regulated (I/R models vs. controls)	↑PART1	Guo et al. (2021)
			I/R injury alleviation	
			↑left ventricular EF and FS, ↓infract size, ↓Bax, ↓cytochrome-c, ↑Bcl-2	
Parkinson’s disease (PD)	C57BL/6 mice (*in vivo* PD model through receiving MPTP)/10 for each group	Down-regulated (MPTP group vs. controls)	PART1 alleviates MPP(+)-associated neuronal damage through modulation of miR-106b-5p/MCL1 axis	Shen et al. (2021)

### Studies in clinical samples

Experiments in clinical samples have shown down-regulation of PART1 in biological samples obtained from patients with Alzheimer’s disease (Huaying et al., 2020), Parkinson’s disease (Chi et al., 2019) and preeclampsia (Peñailillo et al., 2022). On the other hand, PART1 has been found to be up-regulated in nucleus pulposus samples of patients with intervertebral disc degeneration (Gao et al., 2020). Table 10 shows the results of studies on humans samples to ascertain how PART1 is expressed in non-cancerous disorders.

**TABLE 10:** Studies on humans samples to ascertain how PART1 is expressed in non-cancerous disorders (NP: nucleus pulposus).

Disorder	Samples	Expression (disease group vs. normal controls)	References
Alzheimer’s disease (AD)	AD and normal serum samples	Down-regulated	Huaying et al. (2020)
Intervertebral Disc Degeneration (IDD)	30 NP tissues from IDD patients and 30 control NP tissues	Up-regulated	Gao et al. (2020)
Osteoarthritis (OA)	30 OA cartilage tissues and 30 normal cartilage tissues	Down-regulated	Lu et al. (2019)
	35 cartilage tissues from OA patients and 15 cartilage tissues from patients without OA)	Up-regulated	Zhu and Jiang (2019)
Parkinson’s disease (PD)	50 PD blood samples and 22 controls	Down-regulated	Chi et al. (2019)
Preeclampsia	7 preeclampsia placentas and 7 control placenta samples	Down-regulated	Peñailillo et al. (2022)

## Discussion

PART1 is an lncRNA with diverse functions in the carcinogenesis (Lin et al., 2000). It can affect maintenance of cancer stem cells (Cruickshank et al., 2021) and epithelial to mesenchymal transition (Lou et al., 2020) in a variety of tissues. Moreover, it has a role in modulation of response of cancer cells to cisplatin, erlotinib and gefitinib. Mechanistically, PART1 can act as molecular sponge for a variety of miRNAs such as miR-4516, miR-150-5p, miR-143, miR-18a-5p, miR-129, miR-190a-3p, miR-374b, miR-149-5p, miR-590-3p, miR-372-3p, miR-3529-3p, miR-185-5p, miR-17-5p, miR-503-5p, miR-6884-5p, miR-512-3p, miR-122 and miR-503-5p. It can regulate activity of some cancer-related signaling pathways such as Wnt/β-catenin, PI3K/AKT, PTEN and JAK/STAT3 (Zhu et al., 2019).

Transcription of PART1 can be regulated by a number of transcription factors such as androgens, ∆Np63α, FOXP2, STAT1 and YY1. However, the importance of methylation marks in its promotor on PART1 expression has not been elucidated.

An important feature of PART1 participation in the carcinogenesis is its diverse roles and possibly its tissue-dependent functions in this process. Future studies should identify the mechanism of such tissue-dependent functions and determinants its oncogenic *versus* tumor suppressor roles.

Since dysregulation of PART1 in tumor tissues has been associated with aggressive behavior of cancer cells, PART1 can be regarded as a prognostic factor in different types of cancers. However, data regarding the application of PART1 as a diagnostic tool in cancer is not sufficient. Since abnormal expression of PART1 has been reported in a variety of cancers, it is possible that expression levels of PART1 can differentiate cancerous tissues from normal counterparts with appropriate diagnostic power.

Taken together, PART1 participates in the pathogenesis of cancer and a variety of non-cancerous conditions including neurodegenerative disorders. Diagnostic value of PART1 has been assessed in few types of cancers, including esophageal (Kang et al., 2018) and lung (Cao et al., 2021) cancers revealing promising results. Moreover, modulation of expression of PART1 in cancer cell lines or animal models of cancers have been associated with therapeutic benefits. However, this filed lacks sufficient data from clinical models. Future functional studies can provide important information about the underlying mechanisms and consequences of PART1 dysregulation in these disorders. The results of such studies can help in design of novel therapeutic modalities based on this lncRNA, particularly in cancers.

## References

[B1] Cao Y. Zhang R. Luo X. Yang Y. (2021). LncRNA PART1 promotes lung squamous cell carcinoma progression via miR-185-5p/Six1 axis. Hum. Exp. Toxicol. 40 (6), 960–976. PubMed PMID: 33300377. Epub 2020/12/11. eng. 10.1177/0960327120979032 33300377

[B2] Chen S. C. Diao Y. Z. Zhao Z. H. Li X. L. (2020). Inhibition of lncRNA PART1 chemosensitizes wild type but not KRAS mutant NSCLC cells. Cancer Manag. Res. 12, 4453–4460. PubMed PMID: 32606939. Pubmed Central PMCID: PMC7293907. Epub 2020/07/02. eng. 10.2147/CMAR.S245257 32606939 PMC7293907

[B3] Chen Y. Zhou X. Huang C. Li L. Qin Y. Tian Z. (2021). LncRNA PART1 promotes cell proliferation and progression in non-small-cell lung cancer cells via sponging miR-17-5p. J. Cell. Biochem. 122 (3-4), 315–325. PubMed PMID: 33368623. Epub 2020/12/29. eng. 10.1002/jcb.29714 33368623

[B4] Chi L. M. Wang L. P. Jiao D. (2019). Identification of differentially expressed genes and long noncoding RNAs associated with Parkinson's disease. Park. Dis. 2019, 6078251. PubMed PMID: 30867898. Pubmed Central PMCID: PMC6379850. Epub 2019/03/15. eng. 10.1155/2019/6078251 PMC6379850 30867898

[B5] Cruickshank B. M. Wasson M. D. Brown J. M. Fernando W. Venkatesh J. Walker O. L. (2021). LncRNA PART1 promotes proliferation and migration, is associated with cancer stem cells, and alters the miRNA landscape in triple-negative breast cancer. Cancers (Basel) 13 (11), 2644. PubMed PMID: 34072264. Pubmed Central PMCID: PMC8198907. Epub 2021/06/03. eng. 10.3390/cancers13112644 34072264 PMC8198907

[B6] Deng Y. W. Shu Y. G. Sun S. L. (2022). LncRNA PART1 inhibits glioma proliferation and migration via miR-374b/SALL1 axis. Neurochem. Int. 157, 105347. PubMed PMID: 35490895. Epub 2022/05/02. eng. 10.1016/j.neuint.2022.105347 35490895

[B7] Derrien T. Johnson R. Bussotti G. Tanzer A. Djebali S. Tilgner H. (2012). The GENCODE v7 catalog of human long noncoding RNAs: Analysis of their gene structure, evolution, and expression. Genome Res. 22 (9), 1775–1789. 10.1101/gr.132159.111 22955988 PMC3431493

[B8] Gao D. Hao L. Zhao Z. (2020). Long non-coding RNA PART1 promotes intervertebral disc degeneration through regulating the miR-93/MMP2 pathway in nucleus pulposus cells. Int. J. Mol. Med. 46 (1), 289–299. PubMed PMID: 32319551. Pubmed Central PMCID: PMC7255469. Epub 2020/04/23. eng. 10.3892/ijmm.2020.4580 32319551 PMC7255469

[B9] Ghafouri-Fard S. Hussen B. M. Gharebaghi A. Eghtedarian R. Taheri M. (2021). LncRNA signature in colorectal cancer. Pathology-Research Pract. 222, 153432. 10.1016/j.prp.2021.153432 33857856

[B10] Ghafouri-Fard S. Sohrabi B. Hussen B. M. Mehravaran E. Jamali E. Arsang-Jang S. (2022). Down-regulation of MEG3, PANDA and CASC2 as p53-related lncRNAs in breast cancer. Breast Dis. 41 (1), 137–143. PubMed PMID: 35034894. eng. 10.3233/BD-210069 35034894

[B11] Gu W. Ren J. H. Zheng X. Hu X. Y. Hu M. J. (2019). Comprehensive analysis of expression profiles of long non-coding RNAs with associated ceRNA network involved in gastric cancer progression. Mol. Med. Rep. 20 (3), 2209–2218. PubMed PMID: 31322220. Pubmed Central PMCID: PMC6691204. Epub 2019/07/20. eng. 10.3892/mmr.2019.10478 31322220 PMC6691204

[B12] Guo Z. Zhao M. Jia G. Ma R. Li M. (2021). LncRNA PART1 alleviated myocardial ischemia/reperfusion injury via suppressing miR-503-5p/BIRC5 mediated mitochondrial apoptosis. Int. J. Cardiol. 338, 176–184. PubMed PMID: 34082009. Epub 2021/06/04. eng. 10.1016/j.ijcard.2021.05.044 34082009

[B13] Han H. Wang S. Meng J. Lyu G. Ding G. Hu Y. (2020). Long noncoding RNA PART1 restrains aggressive gastric cancer through the epigenetic silencing of PDGFB via the PLZF-mediated recruitment of EZH2. Oncogene 39 (42), 6513–6528. PubMed PMID: 32901105. Epub 2020/09/10. eng. 10.1038/s41388-020-01442-5 32901105

[B14] Hu Y. Ma Z. He Y. Liu W. Su Y. Tang Z. (2017). PART-1 functions as a competitive endogenous RNA for promoting tumor progression by sponging miR-143 in colorectal cancer. Biochem. Biophys. Res. Commun. 490 (2), 317–323. PubMed PMID: 28619512. Epub 2017/06/18. eng. 10.1016/j.bbrc.2017.06.042 28619512

[B15] Hu X. Feng H. Huang H. Gu W. Fang Q. Xie Y. (2019). Downregulated long noncoding RNA PART1 inhibits proliferation and promotes apoptosis in bladder cancer. Technol. Cancer Res. Treat. 18, 1533033819846638. PubMed PMID: 31311442. Pubmed Central PMCID: PMC6636221. Epub 2019/07/18. eng. 10.1177/1533033819846638 31311442 PMC6636221

[B16] Huaying C. Xing J. Luya J. Linhui N. Di S. Xianjun D. (2020). A signature of five long non-coding RNAs for predicting the prognosis of Alzheimer's disease based on competing endogenous RNA networks. Front. Aging Neurosci. 12, 598606. PubMed PMID: 33584243. Pubmed Central PMCID: PMC7876075. Epub 2021/02/16. eng. 10.3389/fnagi.2020.598606 33584243 PMC7876075

[B17] Hussen B. M. Kheder R. K. Abdullah S. T. Hidayat H. J. Rahman H. S. Salihi A. (20222022). Functional interplay between long non-coding RNAs and Breast CSCs. Cancer Cell. Int. 22 (1), 233. 10.1186/s12935-022-02653-4 PMC9306174 35864503

[B18] Jiang M. C. Ni J. J. Cui W. Y. Wang B. Y. Zhuo W. (2019). Emerging roles of lncRNA in cancer and therapeutic opportunities. Am. J. Cancer Res. 9 (7), 1354–1366. PubMed PMID: 31392074. Pubmed Central PMCID: PMC6682721. Epub 2019/08/09. eng. 31392074 PMC6682721

[B19] Jiang Z. Cheng P. Luo B. Huang J. (2020). Construction and analysis of a long non-coding RNA-associated competing endogenous RNA network identified potential prognostic biomarkers in luminal breast cancer. Onco Targets Ther. 13, 4271–4282. PubMed PMID: 32547061. Pubmed Central PMCID: PMC7244246. Epub 2020/06/18. eng. 10.2147/OTT.S240973 32547061 PMC7244246

[B20] Jin Z. Piao L. Sun G. Lv C. Jing Y. Jin R. (2020). Long non-coding RNA PART1 exerts tumor suppressive functions in glioma via sponging miR-190a-3p and inactivation of PTEN/AKT pathway. Onco Targets Ther. 13, 1073–1086. PubMed PMID: 32099409. Pubmed Central PMCID: PMC7007780. Epub 2020/02/27. eng. 10.2147/OTT.S232848 32099409 PMC7007780

[B21] Kang M. Ren M. Li Y. Fu Y. Deng M. Li C. (2018). Exosome-mediated transfer of lncRNA PART1 induces gefitinib resistance in esophageal squamous cell carcinoma via functioning as a competing endogenous RNA. J. Exp. Clin. Cancer Res. 37 (1), 171. PubMed PMID: 30049286. Pubmed Central PMCID: PMC6063009. Epub 2018/07/28. eng. 10.1186/s13046-018-0845-9 30049286 PMC6063009

[B22] Laha S. Saha C. Dutta S. Basu M. Chatterjee R. Ghosh S. (2021). *In silico* analysis of altered expression of long non-coding RNA in SARS-CoV-2 infected cells and their possible regulation by STAT1, STAT3 and interferon regulatory factors. Heliyon 7 (3), e06395. PubMed PMID: 33688586. Pubmed Central PMCID: PMC7914022. Epub 2021/03/11. eng. 10.1016/j.heliyon.2021.e06395 33688586 PMC7914022

[B23] Li M. Zhang W. Zhang S. Wang C. Lin Y. (2017). PART1 expression is associated with poor prognosis and tumor recurrence in stage I-III non-small cell lung cancer. J. Cancer 8 (10), 1795–1800. PubMed PMID: 28819376. Pubmed Central PMCID: PMC5556642. Epub 2017/08/19. eng. 10.7150/jca.18848 28819376 PMC5556642

[B24] Li B. Lou G. Zhang J. Cao N. Yu X. (2022). Repression of lncRNA PART1 attenuates ovarian cancer cell viability, migration and invasion through the miR-503-5p/FOXK1 axis. BMC Cancer 22 (1), 124. PubMed PMID: 35100978. Pubmed Central PMCID: PMC8802513. Epub 2022/02/02. eng. 10.1186/s12885-021-09005-x 35100978 PMC8802513

[B25] Li H. Lei Y. Li S. Li F. Lei J. (2022). LncRNA PART1 stimulates the development of ovarian cancer by up-regulating RACGAP1 and RRM2. Reprod. Sci. 29 (8), 2224–2235. PubMed PMID: 35553409. Epub 2022/05/14. eng. 10.1007/s43032-022-00905-2 35553409

[B26] Lin B. White J. T. Ferguson C. Bumgarner R. Friedman C. Trask B. (2000). PART-1: A novel human prostate-specific, androgen-regulated gene that maps to chromosome 5q12. Cancer Res. 60 (4), 858–863. PubMed PMID: 10706094. Epub 2000/03/08. eng. 10706094

[B27] Liu B. Ma T. Li Q. Wang S. Sun W. Li W. (2019). Identification of a lncRNA-associated competing endogenous RNA-regulated network in clear cell renal cell carcinoma. Mol. Med. Rep. 20 (1), 485–494. PubMed PMID: 31180525. Pubmed Central PMCID: PMC6580006. Epub 2019/06/11. eng. 10.3892/mmr.2019.10290 31180525 PMC6580006

[B28] Liu H. Zhu C. Xu Z. Wang J. Qian L. Zhou Q. (2020). lncRNA PART1 and MIR17HG as ΔNp63α direct targets regulate tumor progression of cervical squamous cell carcinoma. Cancer Sci. 111 (11), 4129–4141. PubMed PMID: 32920922. Pubmed Central PMCID: PMC7648017. Epub 2020/09/14. eng. 10.1111/cas.14649 32920922 PMC7648017

[B29] Lou T. Ke K. Zhang L. Miao C. Liu Y. (2020). LncRNA PART1 facilitates the malignant progression of colorectal cancer via miR-150-5p/LRG1 axis. J. Cell. Biochem. 121 (10), 4271–4281. PubMed PMID: 31898365. Epub 2020/01/04. eng. 10.1002/jcb.29635 31898365

[B30] Lu C. Li Z. Hu S. Cai Y. Peng K. (2019). LncRNA PART-1 targets TGFBR2/Smad3 to regulate cell viability and apoptosis of chondrocytes via acting as miR-590-3p sponge in osteoarthritis. J. Cell. Mol. Med. 23 (12), 8196–8205. PubMed PMID: 31571401. Pubmed Central PMCID: PMC6850963. Epub 2019/10/02. eng. 10.1111/jcmm.14690 31571401 PMC6850963

[B31] Lu S. Y. Hua J. Liu J. Wei M. Y. Liang C. Meng Q. C. (2022). Construction of a paclitaxel-related competitive endogenous RNA network and identification of a potential regulatory axis in pancreatic cancer. Transl. Oncol. 20, 101419. PubMed PMID: 35413498. Pubmed Central PMCID: PMC9018166. Epub 2022/04/13. eng. 10.1016/j.tranon.2022.101419 35413498 PMC9018166

[B32] Lv Y. Wei W. Huang Z. Chen Z. Fang Y. Pan L. (2018). Long non-coding RNA expression profile can predict early recurrence in hepatocellular carcinoma after curative resection. Hepatol. Res. 48 (13), 1140–1148. PubMed PMID: 29924905. Epub 2018/06/21. eng. 10.1111/hepr.13220 29924905

[B33] Marchese F. P. Raimondi I. Huarte M. (2017). The multidimensional mechanisms of long noncoding RNA function. Genome Biol. 18 (1), 206–213. 10.1186/s13059-017-1348-2 29084573 PMC5663108

[B34] Peñailillo R. Monteiro L. J. Acuña-Gallardo S. García F. Velásquez V. Correa P. (2022). Identification of LOC101927355 as a novel biomarker for preeclampsia. Biomedicines 10 (6), 1253. PubMed PMID: 35740273. Pubmed Central PMCID: PMC9219905. Epub 2022/06/25. eng. 10.3390/biomedicines10061253 35740273 PMC9219905

[B35] Pu J. Tan C. Shao Z. Wu X. Zhang Y. Xu Z. (2020). Long noncoding RNA PART1 promotes hepatocellular carcinoma progression via targeting miR-590-3p/HMGB2 Axis. Onco Targets Ther. 13, 9203–9211. PubMed PMID: 32982307. Pubmed Central PMCID: PMC7502387. Epub 2020/09/29. eng. 10.2147/OTT.S259962 32982307 PMC7502387

[B36] Ran R. Gong C. Y. Wang Z. Q. Zhou W. M. Zhang S. B. Shi Y. Q. (2022). Long non-coding RNA PART1: Dual role in cancer. Hum. Cell. 35 (5), 1364–1374. PubMed PMID: 35864416. Epub 2022/07/22. eng. 10.1007/s13577-022-00752-y 35864416

[B37] Shen Y. Cui X. Xu N. Hu Y. Zhang Z. (2021). lncRNA PART1 mitigates MPP(+)-induced neuronal injury in SH-SY5Y cells via micRNA-106b-5p/MCL1 axis. Am. J. Transl. Res. 13 (8), 8897–8908. PubMed PMID: 34540003. Pubmed Central PMCID: PMC8430160. Epub 2021/09/21. eng. 34540003 PMC8430160

[B38] Sidiropoulos M. Chang A. Jung K. Diamandis E. P. (2001). Expression and regulation of prostate androgen regulated transcript-1 (PART-1) and identification of differential expression in prostatic cancer. Br. J. Cancer 85 (3), 393–397. PubMed PMID: 11487271. Pubmed Central PMCID: PMC2364080. Epub 2001/08/07. eng. 10.1054/bjoc.2001.1883 11487271 PMC2364080

[B39] Song Y. Pan Y. Liu J. (2019). Functional analysis of lncRNAs based on competitive endogenous RNA in tongue squamous cell carcinoma. PeerJ 7, e6991. PubMed PMID: 31179185. Pubmed Central PMCID: PMC6544013. Epub 2019/06/11. eng. 10.7717/peerj.6991 31179185 PMC6544013

[B40] Sun M. Geng D. Li S. Chen Z. Zhao W. (2018). LncRNA PART1 modulates toll-like receptor pathways to influence cell proliferation and apoptosis in prostate cancer cells. Biol. Chem. 399 (4), 387–395. PubMed PMID: 29261512. Epub 2017/12/21. eng. 10.1515/hsz-2017-0255 29261512

[B41] Wang Z. Xu R. (2020). lncRNA PART1 promotes breast cancer cell progression by directly targeting miR-4516. Cancer Manag. Res. 12, 7753–7760. PubMed PMID: 32922076. Pubmed Central PMCID: PMC7457826. Epub 2020/09/15. eng. 10.2147/CMAR.S249296 32922076 PMC7457826

[B42] Weng Z. Peng J. Wu W. Zhang C. Zhao J. Gao H. (2021). Downregulation of PART1 inhibits proliferation and differentiation of Hep3B cells by targeting hsa-miR-3529-3p/FOXC2 Axis. J. Oncol. 2021, 7792223. PubMed PMID: 34484336. Pubmed Central PMCID: PMC8410447. Epub 2021/09/07. eng. 10.1155/2021/7792223 34484336 PMC8410447

[B43] Xiao Y. Yang Y. Wang Y. Li X. Wang W. (2019). Five novel genes related to the pathogenesis and progression of pancreatic neuroendocrine tumors by bioinformatics analysis with RT-qPCR verification. Front. Neurosci. 13, 937. PubMed PMID: 31607839. Pubmed Central PMCID: PMC6771308. Epub 2019/10/15. eng. 10.3389/fnins.2019.00937 31607839 PMC6771308

[B44] Yang H. Zhang X. Zhu L. Yang Y. Yin X. (2021). YY1-Induced lncRNA PART1 enhanced resistance of ovarian cancer cells to cisplatin by regulating miR-512-3p/CHRAC1 Axis. DNA Cell. Biol. 40 (6), 821–832. PubMed PMID: 34030482. Epub 2021/05/26. eng. 10.1089/dna.2021.0059 34030482

[B45] Yang L. Lu P. Yang X. Li K. Chen X. Qu S. (2021). Excavating novel diagnostic and prognostic long non-coding RNAs (lncRNAs) for head and neck squamous cell carcinoma: An integrated bioinformatics analysis of competing endogenous RNAs (ceRNAs) and gene co-expression networks. Bioengineered 12 (2), 12821–12838. PubMed PMID: 34898376. Pubmed Central PMCID: PMC8810019. Epub 2021/12/14. eng. 10.1080/21655979.2021.2003925 34898376 PMC8810019

[B46] Yang Z. Gong W. Zhang T. Gao H. (2021). Molecular features of glioma determined and validated using combined TCGA and GTEx data analyses. Front. Oncol. 11, 729137. PubMed PMID: 34660294. Pubmed Central PMCID: PMC8516354. Epub 2021/10/19. eng. 10.3389/fonc.2021.729137 34660294 PMC8516354

[B47] Yu Q. Du Y. Wang S. Zheng X. (2021). LncRNA PART1 promotes cell proliferation and inhibits apoptosis of oral squamous cell carcinoma by blocking EZH2 degradation. J. Biochem. 169 (6), 721–730. PubMed PMID: 33725092. Epub 2021/03/17. eng. 10.1093/jb/mvab026 33725092

[B48] Zhang X. Wang W. Zhu W. Dong J. Cheng Y. Yin Z. (2019). Mechanisms and functions of long non-coding RNAs at multiple regulatory levels. Int. J. Mol. Sci. 20 (22), 5573. PubMed PMID: 31717266. Pubmed Central PMCID: PMC6888083. Epub 2019/11/14. eng. 10.3390/ijms20225573 31717266 PMC6888083

[B49] Zhang S. Cao R. Li Q. Yao M. Chen Y. Zhou H. (2019). Comprehensive analysis of lncRNA-associated competing endogenous RNA network in tongue squamous cell carcinoma. PeerJ 7, e6397. PubMed PMID: 30755833. Pubmed Central PMCID: PMC6368841. Epub 2019/02/14. eng. 10.7717/peerj.6397 30755833 PMC6368841

[B50] Zhang Z. Huo Y. Zhou Z. Zhang P. Hu J. (2021). Role of lncRNA PART1 in intervertebral disc degeneration and associated underlying mechanism. Exp. Ther. Med. 21 (2), 131. PubMed PMID: 33376513. Pubmed Central PMCID: PMC7751492. Epub 2020/12/31. eng. 10.3892/etm.2020.9563 33376513 PMC7751492

[B51] Zhang J. Xu N. Yu C. Miao K. Wang Q. (2021). LncRNA PART1/miR-185-5p/RUNX3 feedback loop modulates osteogenic differentiation of bone marrow mesenchymal stem cells. Autoimmunity 54 (7), 422–429. PubMed PMID: 34431433. Epub 2021/08/26. eng. 10.1080/08916934.2021.1966771 34431433

[B52] Zhao Y. Zhang Q. Liu H. Wang N. Zhang X. Yang S. (2021). lncRNA PART1, manipulated by transcriptional factor FOXP2, suppresses proliferation and invasion in ESCC by regulating the miR-18a-5p/SOX6 signaling axis. Oncol. Rep. 45 (3), 1118–1132. PubMed PMID: 33432363. Pubmed Central PMCID: PMC7859983. Epub 2021/01/13. eng. 10.3892/or.2021.7931 33432363 PMC7859983

[B53] Zhou T. Wu L. Ma N. Tang F. Zong Z. Chen S. (2020). LncRNA PART1 regulates colorectal cancer via targeting miR-150-5p/miR-520h/CTNNB1 and activating Wnt/β-catenin pathway. Int. J. Biochem. Cell. Biol. 118, 105637. PubMed PMID: 31669140. Epub 2019/11/02. eng. 10.1016/j.biocel.2019.105637 31669140

[B54] Zhou C. Wang P. Tu M. Huang Y. Xiong F. Wu Y. (2020). Long non-coding RNA PART1 promotes proliferation, migration and invasion of hepatocellular carcinoma cells via miR-149-5p/MAP2K1 Axis. Cancer Manag. Res. 12, 3771–3782. PubMed PMID: 32547213. Pubmed Central PMCID: PMC7248804. Epub 2020/06/18. eng. 10.2147/CMAR.S246311 32547213 PMC7248804

[B55] Zhou J. Che J. Xu L. Yang W. Zhou W. Zhou C. (2022). Tumor-derived extracellular vesicles containing long noncoding RNA PART1 exert oncogenic effect in hepatocellular carcinoma by polarizing macrophages into M2. Dig. Liver Dis. 54 (4), 543–553. PubMed PMID: 34497040. Epub 2021/09/10. eng. 10.1016/j.dld.2021.07.005 34497040

[B56] Zhu D. Yu Y. Wang W. Wu K. Liu D. Yang Y. (2019). Long noncoding RNA PART1 promotes progression of non-small cell lung cancer cells via JAK-STAT signaling pathway. Cancer Med. 8 (13), 6064–6081. PubMed PMID: 31436388. Pubmed Central PMCID: PMC6792487. Epub 2019/08/23. eng. 10.1002/cam4.2494 31436388 PMC6792487

[B57] Zhu Y. J. Jiang D. M. (2019). LncRNA PART1 modulates chondrocyte proliferation, apoptosis, and extracellular matrix degradation in osteoarthritis via regulating miR-373-3p/SOX4 axis. Eur. Rev. Med. Pharmacol. Sci. 23 (19), 8175–8185. PubMed PMID: 31646607. Epub 2019/10/28. eng. 10.26355/eurrev_201910_19124 31646607

